# Wireless High Temperature Sensing Chipless Tag Based on a Diamond Ring Resonator

**DOI:** 10.3390/mi14040731

**Published:** 2023-03-25

**Authors:** Bo Wang, Youwei Li, Tingting Gu, Ke Wang

**Affiliations:** 1 School of Automation, Xi’an University of Posts and Telecommunications, Xi’an 710121, China; 2 Department of Smart New Energy, GuiYang Engineering Corporation Limited, Guiyang 550081, China

**Keywords:** double diamond split rings resonator, passive wireless, high temperature sensor, alumina ceramic, high sensitivity

## Abstract

A passive wireless sensor is designed for real-time monitoring of a high temperature environment. The sensor is composed of a double diamond split rings resonant structure and an alumina ceramic substrate with a size of 23 × 23 × 0.5 mm^3^. The alumina ceramic substrate is selected as the temperature sensing material. The principle is that the permittivity of the alumina ceramic changes with the temperature and the resonant frequency of the sensor shifts accordingly. Its permittivity bridges the relation between the temperature and resonant frequency. Therefore, real time temperatures can be measured by monitoring the resonant frequency. The simulation results show that the designed sensor can monitor temperatures in the range 200~1000 °C corresponding to a resonant frequency of 6.79~6.49 GHz with shifting 300 MHz and a sensitivity of 0.375 MHz/°C, and demonstrate the quasi-linear relation between resonant frequency and temperature. The sensor has the advantages of wide temperature range, good sensitivity, low cost and small size, which gives it superiority in high temperature applications.

## 1. Introduction

Expensive workpieces, such as components of aircraft, thermal reactors and gasifier parts, are easily damaged at high temperatures [1]. The aerospace and manufacturing industries in particular have a great demand for high performance temperature sensors to monitor harsh environments, where turbine engine blades can deform at high temperatures when they are running at high speeds. Real-time monitoring of the temperature of workpieces helps to give advance warning and enables replacement of workpieces to prevent wider damage to the equipment. The traditional method is to use infrared thermocouples [2,3] or thermal imagers [4,5,6] for remote temperature monitoring, but their limitations are visual range and the need for a power supply. In order to overcome these limitations, a chipless sensor without visualization and passive in nature is proposed. In recent years, the flexibility, low manufacturing cost and new structure of sensors have changed the patterns of sensor use [7]. Reference [8] describes a temperature sensor and wireless sensor system based on surface acoustic waves (SAWs) for real-time monitoring of wafer surface temperature in the plasma chamber. The antenna size is 250 × 197 × 1.524 mm^3^. Because SAWs use a specific piezoelectric crystal, the signal is susceptible to noise interference and the operating temperature is greatly limited [9,10,11]. References [12,13,14,15,16] integrate the sensing function into the chip, which gives it the characteristics of high precision and low power consumption. In view of the high cost of electronic chips, their wide use is restricted. At the same time, chip-based sensor tags are limited by the maximum operating temperature of 150 °C.

The temperature sensor proposed is based on a microwave dielectric resonator using alumina ceramic as the substrate [17]. An etched rectangular hole is located on the surface of the temperature sensor. On the one hand, it acts as an excitation device. On the other hand, it acts as a coupling device. The overall size is 29 × 29 × 5 mm^3^ and the operating temperature range is 27~800 °C. Reference [18] describes a passive wireless LC resonant sensor based on DuPont 951 ceramics. The sensor is utilized in a harsh environment of high temperature and pressure and the measuring temperature range is 50~400 °C with a sensitivity of 0.24 MHz/°C. Reference [19] describes a high temperature sensor based on a split ring resonator. The sensor is fabricated on alumina ceramic substrate by screen printing. The temperature range of the sensor is 28~1100 °C; however, its sensitivity is only 95.63 kHz/°C. Reference [20] describes a new type of high temperature sensor with an inductance and capacitance separation structure, which is composed of aluminum oxide ceramic and platinum metal. The working temperature of the sensor is as high as 1400 °C with a sensitivity of 6.7562 kHz/°C. The size of the substrate is 75 × 15 × 0.6 mm^3^.

At present, the sensors working in the microwave frequency band mainly adopt a wireless sensing technology based on LC resonance. However, when the sensor is affixed to the surface of metal objects, the generated eddy current seriously affects the measurement results; this has restricted the further development of the technology based on LC resonance [21]. Split Ring Resonators (SRRs) have the advantages of easy adjustment of resonant frequency, simple design and planar structure [22,23,24]. Researchers have carried out numerous studies. The structure of split ring resonator was first proposed by Pendy and others [25]. Smith et al. demonstrated the unique electromagnetic response of the split ring structure through a large number of experiments. The resonant frequency of a chipless RFID sensor can be adjusted within a given frequency band by changing the shape [26,27] and size of the tag [28]. Resonant frequency response is the key parameter of chipless RFID sensors, because it enables the direct monitoring of the variation of the environmental parameters.

To sum up, the passive wireless temperature sensors have some problems, such as large size, poor high temperature resistance and narrow working range. Based on the principle of electromagnetic backscattering and the inherent temperature dependence of the relative dielectric constant of alumina ceramic, a new sensor is proposed in this paper to improve the operating temperature limitation of the sensor. The double diamond split rings structure is applied to the temperature sensor, which has good sensitivity, simple fabrication and flexible design. The overall size is 23 × 23 × 0.5 mm^3^ and the sensitivity is 0.375 MHz/°C. The measuring temperature range is 200~1000 °C.

## 2. Sensor Design

### 2.1. Temperature Sensor Structure

**Figure 1:**
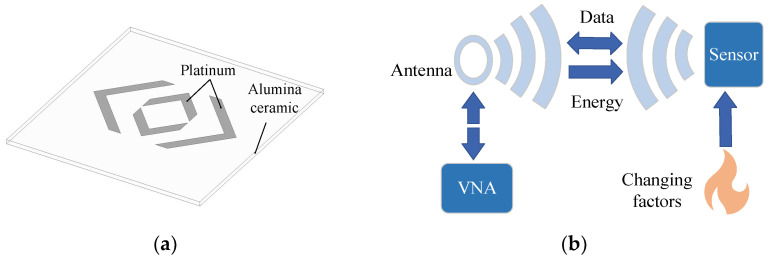
(**a**) Side view. (**b**) Working principle diagram.

Figure 1a shows a side view of the passive wireless temperature sensor with a double diamond split rings structure printed with platinum on the surface of an alumina ceramic substrate. As a temperature sensitive material, the alumina ceramic has dielectric constant -temperature characteristics and high temperature resistance [29]. Figure 1b shows the working principle of the temperature sensor. The sensor, inquiry antenna and vector network analyzer (VNA) constitute the three key parts of the system. When the electromagnetic wave excites the sensor, its structure couples with the electromagnetic wave and reflects the parameters of the sensor to the antenna. The alumina ceramic shows different dielectric constants at different temperatures. The insertion loss (S_21_) of the electromagnetic waves is obtained by the VNA, which reveals the internal relationship between the resonant frequency of the sensor and the change of the ambient temperature. It is analyzed to realize the detection function of the ambient temperature.

### 2.2. Double Diamond Split Rings Resonator

**Figure 2:**
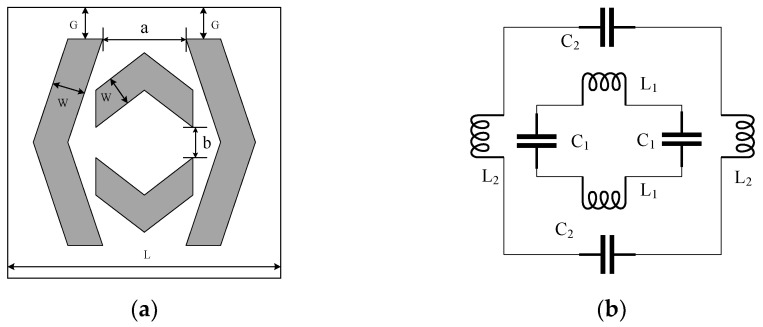
(**a**) Top view of temperature sensor. (**b**) Equivalent circuit diagram of double diamond split rings resonator.

There are two main reasons for selecting double diamond split rings as the resonant structure of the temperature sensor [30]. In terms of characteristics, split ring resonators have good RCS frequency response, high Q value and good resonant characteristics. In terms of structure, the design is simple and symmetrical. The equivalent capacitance and inductance of the sensor can be adjusted by changing the split length and line width of the diamond structure. The top view of the temperature sensor is shown in Figure 2a, where the length of the substrate *L* = 23 mm, the diamond width *W* = 1 mm, the gap between diamond structure and the edge of the substrate *G* = 6.5 mm, the split of the outer ring *a* = 4.14 mm, and the split of the inner ring *b* = 0.34 mm. For the double rings resonant structure, the coupling capacitance between the rings will be destroyed by the split structure. Because the coupling capacitance between the rings disappears, the sensor will show the dual-band characteristics. Figure 2b shows the equivalent circuit diagram of the double diamond split rings resonator. *C*_1_ is the split capacitance of the inner ring, *L*_1_ is the split inductance of the inner ring, *C*_2_ is the split capacitance of the outer ring, and *L*_2_ is the split inductance of the outer ring. The inner ring resonance is $f_{1}=(2\pi \sqrt{L_{a}C_{a}}{)}^{-1}$, including $L_{a}=2L_{1}$, $C_{a}=\frac{C_{1}}{2}.$ The outer ring resonance is $f_{2}=(2\pi \sqrt{L_{b}C_{b}}{)}^{-1}$, including $L_{b}=2L_{2}$, $C_{b}=\frac{C_{2}}{2}$.

(1) $$f_{1}=(2\pi \sqrt{L_{1}C_{1}}{)}^{-1}$$

(2) $$f_{2}=(2\pi \sqrt{L_{2}C_{2}}{)}^{-1}$$

**Figure 3:**
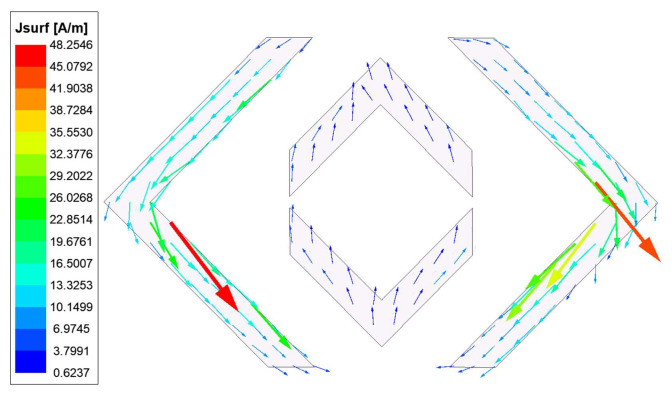
Current distribution of temperature sensor.

When the metal surface is excited by electromagnetic waves, the original metal surface current distribution is changed due to the splits of the double diamond. The current is mainly concentrated at the splits, meaning that the resonance characteristics are better. In the resonance state, the metal surface of the diamond structure has an induced current, and then the induced current produces a magnetic field. The current direction is shown in Figure 3. The surface current of the outer ring structure flows from top to bottom, while the surface current of the inner ring structure flows from bottom to top. It can be seen that the outer diamond structure has large current intensity.

### 2.3. Sensing Material

Sensitivity is one key factor of the sensor tag, and can be realized by use of sensitive material. The passive wireless temperature sensor structure uses two kinds of material. One is the resonant structure surface covered with temperature sensitive material, the other is the dielectric substrate itself as temperature sensitive material. In this paper, alumina ceramic is selected as the substrate of the temperature sensor.

The dielectric constant of the alumina ceramic has a temperature-sensitive property, which changes monotonically with the varying of ambient temperature [31]. It stimulates a shift of the resonant frequency of the sensor. Furthermore, the utilization of alumina ceramic mainly composed of aluminum oxide as the temperature sensor has the advantage that the sensor can operate at very high temperatures due to the material being sintered at temperatures around 2000 °C; however, it still maintains its low thermal expansion, high thermal conductivity and mechanical strength. The sensor’s temperature varies accurately with the environment temperature due to its high thermal conductivity. Meanwhile, the high mechanical strength makes the sensor damage resistant and prolongs the sensor life. The permittivity of the alumina ceramic increases from 9.7 to 11 as temperatures increase from 50 to 1000 °C.

When the sensor is exposed in alternating electromagnetic field, there will be polarized electrons in the alumina ceramic [19]. When the temperature *T* increases gradually, the dielectric constant *ε_r_* of the substrate increases and the thermal motion of electrons becomes more obvious. The regular motion of electrons caused by the electric field decreases and the electron polarization weakens at the same time, so that the actual electric field *E_e_* intensity decreases. The higher the temperature increases, the faster the resonant frequency *f* changes. The relationship between *T* and f can be obtained from reference [19]:

(3) $$T↑\to \varepsilon_{r}↑\to E_{e}↓\to f↓$$

According to Equation (3), the temperature *T* is inversely proportional to the resonant frequency *f*. It shows that the alumina ceramic substrate as a sensitive material can be theoretically be applied in the temperature sensor.

## 3. Simulation Analysis

**Figure 4:**
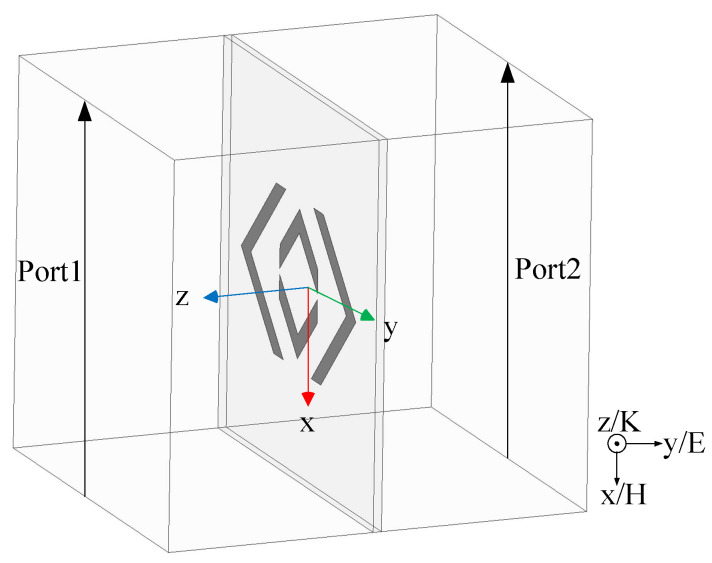
HFSS simulation model of temperature sensor.

The conclusion in Section 2.3 is that the temperature changes the permittivity of alumina ceramic resulting in the resonant frequency of the sensor shifting. Hence, the alumina ceramic is suitable as a sensing substrate. The radio frequency simulation software HFSS was used to model the passive wireless temperature sensor. A structural design optimization and temperature sensing mechanism simulation of alumina ceramic substrate were carried out. In the simulation experiment, the simulated electromagnetic wave propagates along the z axis. The two boundary surfaces of the perfect electrical conductor (PEC) are set on the two planes perpendicular to the y axis and the two boundary surfaces of the perfect magnetic conductor (PMC) are set on the two planes perpendicular to the x axis. The PEC and PMC are perpendicular to each other. The simulation model of the temperature sensor finally constructed is shown in Figure 4.

**Figure 5:**
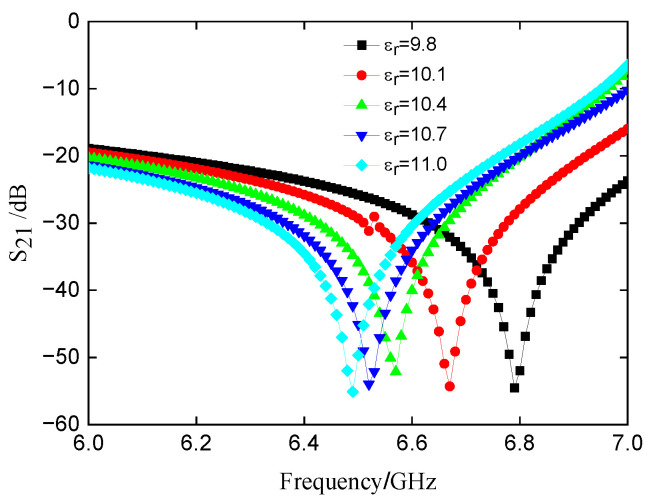
Variation of resonant frequency with different dielectric constants.

Figure 5 shows the relationship between the dielectric constant of the alumina ceramic substrate and the resonant frequency of the sensor, which is obtained by modeling and simulating with the RF software HFSS. Because the dielectric constant of the substrate corresponds to the temperature, the temperature changing process in the environment can be simulated by changing the value of the dielectric constant of the substrate. When the temperature rises from 200 °C to 1000 °C, the dielectric constant of alumina ceramic increases from 9.8 to 11.0 [32]. In the simulation, the dielectric constant of the substrate is set to 9.8, 10.1, 10.4, 10.7 and 11.0 corresponding to the temperatures 200 °C, 400 °C, 600 °C, 800 °C, 1000 °C. The variation of the resonant frequency of the sensor is affected when the temperature rises gradually in the simulation environment.

**Figure 6:**
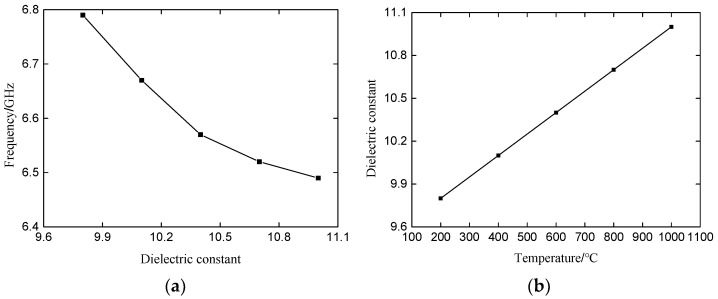
(**a**) Relation between resonant frequency and dielectric constant. (**b**) Relationship between dielectric constant and temperature.

**Figure 7:**
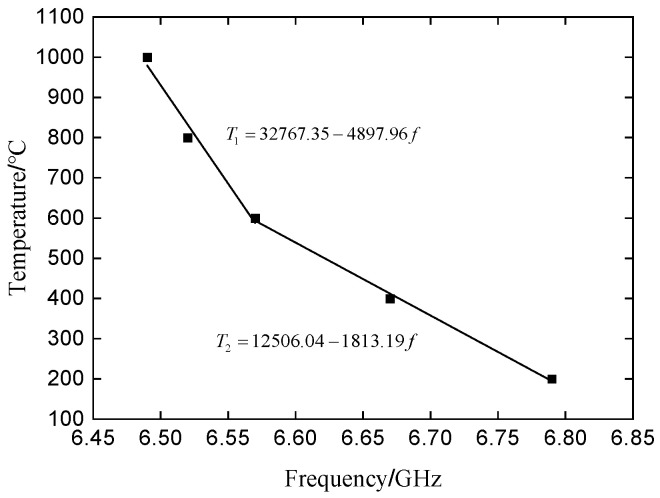
Relation between temperature and resonant frequency.

Figure 6a shows the relationship between the resonant frequency of the sensor and the dielectric constant of the substrate. The resonant frequency points and the dielectric constant are extracted from Figure 5. With the increase of dielectric constant, the resonant frequency decreases gradually. It is known from reference [32] that the dielectric constant of alumina ceramic changes with temperature as shown in Figure 6b. Figure 6a,b can be associated to extract the temperature and resonant frequency relationship shown in Figure 7. In order to characterize the temperature-frequency characteristics of the sensor, the simulation results are fitted in two frequency ranges. The corresponding fitting formulas are selected in different frequency ranges:

(4) $$T_{1}=32767.35-4897.96f(6.49\leq f<6.57)$$

(5) $$T_{2}=12506.04-1813.19f(6.57<f\leq 6.79)$$

where *f* is the resonant frequency, *T*_1_ and *T*_2_ are the corresponding temperature values in different frequency bands. To sum up, the resonant frequency of the sensor and the temperature decrease monotonically, which proves the feasibility of the working principle of the wireless passive sensor. At the same time, it shows that monitoring the resonant frequency of the sensor can provide data on the ambient temperature.

In order to better characterize the response temperature change of the sensor, the concept of relative sensitivity *S* [19] is introduced to compare the temperature response characteristics and the performance of the sensor. The expression is as follows:

(6) $$S=\frac{\left|f-f_{0}\right|}{\left|t-t_{0}\right|}$$

In Equation (6), *f*_0_ and *t*_0_, respectively, represent the initial frequency and temperature, while f and t are in one-to-one correspondence and *f* represents the resonant frequency corresponding to temperature t. Table 1 shows the relative sensitivity of the temperature sensor in the range 200~1000 °C. The relative sensitivity of the sensor can reach 0.375 MHz/°C, which is better than the relative sensitivity of 0.0956 MHz/°C in reference [19].

The parameters in Table 2 compare the temperature sensor based on the ceramic substrate with other temperature sensors using ceramic as sensitive material. The sensitivity of the passive wireless temperature sensor presented in this paper is 0.375 MHz/°C with a smaller size and wider monitoring range. Comparing with references [17,18,19,20] the ceramic substrate area in reference [18] is the smallest, but the working range of the sensor is narrow, from 50 °C to 400 °C. The temperature sensing range of reference [19] is wider, but its area is larger and the sensitivity is 0.0956 MHz/°C. Reference [20] has the widest temperature sensing range, but its sensitivity is only 0.0068 MHz/°C and the substrate size is also large. In conclusion, the temperature sensor proposed in this paper has good comprehensive performance.

## 4. Conclusions

A passive wireless temperature sensor based on the double diamond split rings structure is proposed. The aluminum ceramic substrate is used as the temperature sensitive material and the metal platinum is printed on its surface to realize the simple manufacture of the whole sensor.

(1) The passive wireless high temperature sensor is proposed. The working principle of the sensor is introduced and the equivalent circuit and surface current distribution of double diamond split rings are analyzed.

(2) HFSS software is used to simulate the working principle of the sensor and the fitting curve formulas are obtained. It is proved that the resonant frequency of the sensor changes monotonically with the temperature. They are inversely proportional, that is, the resonant frequency decreases as the temperature increases When the temperature rises from 200 °C to 1000 °C, the total frequency is reduced by 300 MHz.

(3) The temperature sensor adopts a miniaturized design; the overall size is 23 × 23 × 0.5 mm^3^. The maximum sensitivity can reach 0.6 MHz/°C. Compared with the existing sensors, the designed sensor performs better and has the advantages of low cost and simple structure. It can realize the monitoring of high temperature environments.

## Figures and Tables

**Table 1:** 200~1000 °C relative sensitivity *S*.

T/°C	200	400	600	800	1000
S/(MHz/°C)	—	0.6	0.55	0.45	0.375

**Table 2:** Parameter comparison of temperature sensors.

Reference	Fabrication Technology	Materials	Area/cm^2^	Sensitivity/(MHz/°C)	Range/°C
This paper	Screen printing	Alumina ceramic	5.29	0.375	200~1000
[17]	Etching	Alumina ceramic	8.41	0.194	27~800
[18]	LTCC	DuPont ceramics	4.84	0.24	50~400
[19]	Screen printing	Alumina ceramic	6.16	0.0956	28~1100
[20]	Screen printing	Alumina ceramic	10.5	0.0068	43~1500

## Data Availability

The authors confirm that the data supporting the findings of this study are available within the article.
